# Suicide methods in Europe: a gender-specific analysis of countries participating in the “European Alliance Against Depression”

**DOI:** 10.1136/jech.2007.065391

**Published:** 2008-05-13

**Authors:** A Värnik, K Kõlves, C M van der Feltz-Cornelis, A Marusic, H Oskarsson, A Palmer, T Reisch, G Scheerder, E Arensman, E Aromaa, G Giupponi, R Gusmäo, M Maxwell, C Pull, A Szekely, V Pérez Sola, U Hegerl

**Affiliations:** 1 Estonian-Swedish Mental Health and Suicidology Institute, Estonian Centre of Behavioural and Health Sciences, Estonia; 2 Tallinn University, Estonia; 3 Trimbos-Instituut/Utrecht and VU Medical Centre, Institute of Extramural Research, Amsterdam, The Netherlands; 4 University of Primorska, Koper, Slovenia; 5 Directorate of Health Campaign Against Depression and Suicide, Seltjarnarnes, Iceland; 6 Centre for Health Services Studies, University of Kent, UK; 7 University of Bern, Department of Psychiatry, Bern, Switzerland; 8 Katholieke Universiteit Leuven, LUCAS-Centre, Leuven, Belgium; 9 National Suicide Research Foundation, Cork, Ireland; 10 Vaasa Central Hospital, Psychiatric Unit, Vaasa, Finland; 11 S Maurizo Hospital Bolzano, Bozen, Italy; 12 Universidade Nova de Lisboa, Faculdade de Ciências Médicas, Lisbon, Portugal; 13 Department of Applied Social Sciences, University of Stirling, UK; 14 Centre Hospitalier de Luxembourg, Luxembourg, Luxembourg; 15 Semmelweis University Budapest, Institute of Behavioural Sciences, Budapest, Hungary; 16 Hospital de la Santa Creu i Sant Pau, Barcelona, Spain; 17 University of Leipzig, Department of Psychiatry, Leipzig, Germany

## Abstract

**Objective:**

To identify the most frequent gender-specific suicide methods in Europe.

**Design:**

Proportions of seven predominant suicide methods utilised in 16 countries participating in the European Alliance Against Depression (EAAD) were reported in total and cross-nationally. Relative risk (RR) relating to suicide methods and gender was calculated. To group countries by pattern of suicide methods, hierarchical clustering was applied.

**Setting and participants:**

Data on suicide methods for 119 122 male and 41 338 female cases in 2000–4/5 from 16 EAAD countries, covering 52% of European population were obtained.

**Results:**

Hanging was the most prevalent suicide method among both males (54.3%) and females (35.6%). For males, hanging was followed by firearms (9.7%) and poisoning by drugs (8.6%); for females, by poisoning by drugs (24.7%) and jumping from a high place (14.5%). Only in Switzerland did hanging rank as second for males after firearms. Hanging ranked first among females in eight countries, poisoning by drugs in five and jumping from a high place in three. In all countries, males had a higher risk than females of using firearms and hanging and a lower risk of poisoning by drugs, drowning and jumping. Grouping showed that countries might be divided into five main groups among males; for females, grouping did not yield clear results.

**Conclusions:**

Research on suicide methods could lead to the development of gender-specific intervention strategies. Nevertheless, other approaches, such as better identification and treatment of mental disorders and the improvement of toxicological aid should be put in place.

An important task for researchers and public health officials is to seek effective intervention strategies for suicide prevention. Studies about the methods of suicide in relation to different cultural, ethnic, gender and age groups can provide useful information for developing effective prevention and intervention programmes.^1^ A review^2^ examining effectiveness of specific suicide-preventive interventions based on a comprehensive literature survey performed via electronic searches considered only two types of interventions as evidence based: educating physicians to recognise and treat depression and restricting access to lethal means.

Methods that individuals choose in suicide vary widely in their probability of resulting death. At least two general factors determine lethality by a particular method. Firstly, the time span between the initiation of a suicidal act and expected death is crucial for outcome. Highly lethal suicide methods are fairly quick, reducing the possibility of detection and intervention. Lethality is lower if the method chosen gives time for intervention, as well as the possibility of changing one’s mind and seeking help. Also critical for outcome is the availability of medical aid and its quality related to the method used.^3^

Male suicide rates greatly exceeded female rates in all European countries (http://data.euro.who.int/hfamdb/). Several studies have suggested that a primary reason for gender differences in completed suicides is the result of differences in methods used by the two groups.^4^^–^7

A number of factors may influence an individual’s decision regarding method in a suicide act. Hendin^8^ attributes choice of method to a form of final communication of both personal and social needs: a last message. He believes that this message is an expression of the life left behind and the individual’s hopes to resolve the conflicts that plagued him or her in life.

Different methods yield mixed results in terms of lethality. Therefore, there is no single answer to the question whether method choice can be used as a measure of suicidal intent. Similarly, a review of the literature reveals disparate conclusions. Some have found no significant gender differences between the level of suicidal intent,^4^ ^9^ ^10^ others have argued that women tend to use less lethal methods because they have less desire to kill themselves.^3^ ^6^ ^11^

Gender differences in the utilisation of suicide methods may depend on beliefs about culturally acceptable gender-specific self-destructive behaviours. If completed suicide is viewed as a masculine behaviour, then an attempted suicide does not suit the men’s role. Following this rationale, suicidal males could choose violent methods, as it would make them more likely to “succeed” in their action.^9^ Some attribute women’s use of less violent methods to their concern for bodily appearance—how they will look in death.^11^ ^12^

The EAAD (European Alliance Against Depression), an international partnership of 16 European countries, established in 2004, aims to reduce suicide rates in participating countries by implementing evidence-based actions and creating recommendations for effective interventions.^13^ The EAAD intervention concept is partly based on experiences of the Nuremberg Alliance Against Depression, a model project that has been shown to be effective in reducing the number of suicidal acts.^14^ The epidemiological data on methods of suicide gathered in the course of this project provide an opportunity to contribute to the field of suicide prevention.

The present study reports the gender-specific suicide methods in 16 countries participating in the EAAD, compares the results between countries and makes efforts to group countries with similar patterns of suicide methods.

## METHODS

Data were collected from 16 European countries participating in the European Commission funded project “European Alliance Against Depression”. The United Kingdom is represented in the study by two countries: England and Scotland. In Belgium, Estonia, Finland, Germany, Hungary, Iceland, Italy (South Tyrol), Luxembourg, The Netherlands, Portugal, Scotland, Slovenia, Spain and Switzerland, the method used in a suicide act was identified according to the codes X60-X84 by the tenth revision of international statistical classification of diseases and related health problems (ICD-10; World Health Organization, 1992). In the following countries, suicide acts were identified by ICD-9 (World Health Organization, 1978) codes E950-E959 for some or all of the study period: England in 2000, Portugal in 2000–1 and Ireland for the entire study period.

As data on method-specific suicide are not available through the WHO databank, the data were obtained from the appropriate statistics institutions of the EAAD countries. Specifically, the responsible institutions that provided the data are Belgium, Flemish Ministry of Health in cooperation with the National Institute of Statistics (Statistics Belgium); Estonia, Statistics Estonia (Statistikaamet) www.stat.ee; England, South East Public Health Observatory (SEPHO) www.sepho.nhs.uk; Finland, Statistics Finland (Tilastokeskus) www.stat.fi; Germany, Information System of the Federal Health Monitoring (Gesundheitsberichterstattung des Bundes) www.gbe-bund.de; Hungary, Hungarian Central Statistical Office; Iceland, Statistics Iceland www.statice.is; Ireland, Central Statistics Office (CSO) Ireland www.cso.ie; Italy (South Tyrol), Public Health Office of S Maurizo Hospital Bolzano (Servizio Igiene Ospedale S Maurizio Provincia di Bolzano); Luxembourg, (Ministère de la Santé) www.ms.etat.lu; The Netherlands, Statistics Netherlands (Centraal Bureau voor de Statistiek) http://statline.cbs.nl/; Portugal, Statistics Institute (Instituto Nacional de Estatística) http://www.ine.pt/portal/; Scotland, General Register Office for Scotland www.gro-scotland.gov.uk; Slovenia, Institute of Public Health and the Statistical Office of the Republic of Slovenia; Spain, National Statistics Institute (Instituto Nacional de Estatistica) www.ine.es; Switzerland, Swiss Federal Statistical Office (Bundesamt für Statistik) www.bfs.admin.ch.

The EAAD database contains suicide deaths registered with the above mentioned institutions responsible for health statistics of participating countries for the following years: 2000–5 in Estonia, Finland, Germany, Italy (South Tyrol county), Luxembourg, The Netherlands, Scotland and Spain, in 2000–4 in Belgium (Flemish Region, 59% of entire population), England, Hungary, Iceland, Ireland, Portugal, Slovenia and Switzerland. All suicide methods were re-categorised into eight groups using all ICD-10 X-codes (table 1). The category “other” includes methods that accounted for less than 3% of the overall number of suicides: explosive material (X75), fire (X76), hot vapours (X77), cutting/piercing with sharp object (X78), cutting/piercing with blunt object (X79), crashing of motor vehicle (X82), with other specified and classifiable means (X83) and other unspecified means (X84).

**Table 1:** Suicide methods in absolute numbers and percentages by gender in EAAD countries combined through the years 2000–4/5*

	Male		Female		Total	
	No	%	No	%	No	%
Poisoning by drugs (X60–X64)	10 211	8.6	10 209	24.7	20 420	12.7
Poisoning by other means (X65–X69)	6432	5.4	1766	4.3	8198	5.1
Hanging (X70)	64 730	54.3	14 735	35.6	79 465	49.5
Drowning (X71)	3598	3.0	3186	7.7	6784	4.2
Firearms (X72–X74)	11 593	9.7	548	1.3	12 141	7.6
Jumping (X80)	9341	7.8	5977	14.5	15 318	9.5
Moving object (X81)	5817	4.9	2162	5.2	7979	5.0
Other methods	7400	6.2	2755	6.7	10 155	6.3
Total	119 122	100	41 338	100	160 460	100

*Available years summarised.

### Statistical analysis

Statistical analysis was performed with SPSS 14.0 and StatsDirect 2.3.7. To compare male and female suicide methods, relative risks (RR) with 95% confidence intervals in all studied countries were calculated separately and in total. For grouping countries by their distribution of suicide methods, standardised for Z-scores (the Z-score reveals how many units of the standard deviation a case is above or below the mean) separately by gender hierarchical clustering using Ward method was applied. This procedure attempts to identify relatively homogeneous groups of cases (countries) based on selected characteristics (suicide methods), using an algorithm that starts with each case in a separate cluster and combines clusters until only one is left (SPSS 14.0). The level of statistical significance was set at α = 0.05.

## RESULTS

This report is based on 160 460 suicide cases, 119 122 (74%) males and 41 338 (26%) females, who committed suicide in 16 European countries during the years 2000–4/5. The most frequent suicide method for all studied EAAD countries for both genders was hanging (54.3% among male and 35.6% among female “suicidents”). For males, hanging was followed distantly and in almost equal place by firearms (9.7%) and poisoning by drugs (8.6%). For females, poisoning by drugs (24.7%) and jumping from a high place (14.5%) were the next most frequent methods (table 1).

Hanging was the most frequent suicide method among males in all countries except in Switzerland, where firearms ranked first. Firearms were the second most common suicide method among males in Belgium, Estonia, Finland, Germany and Slovenia. Use of firearms ranked lowest in Scotland (table 2).

**Table 2:** Suicide methods in percentages in EAAD countries by gender, annual means for the years 2000–4/5

	Poisoning by drugs		Poisoning by other means		Hanging	Drowning	Firearms	Jumping	Moving object		Other methods
Belgium											
Males	6.8		3.9		57.3	7.0	12.0	3.3	5.2		4.5
Females	17.4		3.3		36.9	18.6	3.7	7.6	6.3		6.1
England											
Males	14.7		11.5		55.4	2.1	3.6	2.7	5.4		4.7
Females	41.0		4.5		36.4	4.5	0.6	3.6	5.9		3.5
Estonia											
Males	1.6		2.4		79.1	0.3	8.5	3.2	0.4		4.4
Females	9.2		2.6		70.8	1.9	1.7	10.6	0.0		3.3
Finland											
Males	17.3		6.1		33.4	3.7	24.6	4.7	4.8		5.4
Females	50.1		1.2		20.7	9.7	2.1	6.6	6.0		3.5
Germany											
Males	8.2		4.1		54.9	2.0	10.4	7.8	5.6		7.1
Females	23.2		3.1		37.9	6.7	1.3	14.1	5.2		8.3
Hungary											
Males	6.9		5.1		69.7	1.2	4.1	5.0	2.6		5.5
Females	26.6		6.6		45.3	3.9	0.6	9.6	2.4		5.0
Iceland											
Males	22.7		8.6		36.7	6.3	15.6	4.7	0.0		5.5
Females	45.2		14.3		16.7	16.7	0.0	2.4	0.0		4.8
Ireland											
Males	8.0		2.9		60.2	16.2	7.6	1.8	2.0		1.2
Females	28.1		1.3		33.4	25.5	1.9	3.6	4.9		1.3
Italy											
Males	2.7		9.5		55.4	5.0	6.8	14.0	1.8		5.0
Females	7.6		7.6		28.8	12.1	1.5	33.3	6.1		3.0
Luxembourg											
Males	8.7		8.0		39.1	2.7	13.0	17.1	2.0		9.4
Females	28.6		8.2		14.3	3.1	1.0	32.7	3.1		9.2
Netherlands											
Males	10.8		3.7		48.4	6.5	4.1	8.2	11.7		6.6
Females	24.4		2.5		33.8	10.4	0.5	10.7	12.0		5.8
Portugal											
Males	2.1		13.2		52.3	4.3	12.5	5.7	1.1		8.7
Females	9.1		21.8		31.5	13.1	2.7	11.7	1.4		8.8
Scotland											
Males	17.4		6.7		56.2	3.9	2.1	5.6	2.5		5.5
Females	46.0		1.1		33.3	6.4	0.1	6.3	1.6		5.1
Slovenia											
Males	2.2		7.3		65.9	2.3	12.6	3.7	1.5		4.6
Females	8.3		5.1		55.4	11.9	1.1	9.4	3.7		5.1
Spain											
Males	3.4		4.4		52.7	3.9	6.9	18.3	3.7		6.7
Females	8.1		6.4		29.5	6.8	1.0	37.4	3.0		7.8
Switzerland											
Males	13.1		3.5		27.2	3.0	33.6	9.2	6.0		4.5
Females	37.5		2.4		19.1	10.0	3.6	14.7	8.5		4.3
Total											
Males	8.6		5.5		54.5	3.0	9.7	7.6	4.8		6.1
Females	24.9		4.4		35.7	7.8	1.3	14.1	5.2		6.6

Hanging was the most common method among females in eight countries: Belgium, Estonia, Germany, Hungary, Ireland, Netherlands, Portugal and Slovenia. Poisoning by drugs was the most frequent method for females in five countries: England, Finland, Iceland, Scotland and Switzerland. Among females, jumping from a high place ranked first in Italy (South Tyrol), Luxembourg and Spain (table 2).

A comparison of male and female suicide methods showed that males have a statistically significantly higher risk than females of using firearms, hanging and poisoning by other means, and lower risk in poisoning by drugs, drowning and jumping (table 3). In all EAAD countries, males had a higher risk of hanging than females. The situation was similar for firearms; only in Italy did the male-female difference not reach statistical significance.

**Table 3:** Relative risks by suicide methods in EAAD countries comparing males to females

	Poisoning by drugs		Poisoning by other means		Hanging	Drowning	Firearms	Jumping	Moving object		Other methods
Belgium											
RR	0.39*		1.2		1.55*	0.38*	3.20*	0.43*	0.83		0.73*
95% CI	0.33 to 0.46		0.87 to 1.62		1.45 to 1.67	0.32 to 0.44	2.45 to 4.19	0.34 to 0.55	0.66 to 1.05		0.57 to 0.93
England											
RR	0.36*		2.54*		1.52*	0.46*	6.39*	0.73*	0.92		1.35*
95% CI	0.34 to 0.38		2.17 to 2.98		1.45 to 1.59	0.38 to 0.56	4.10 to 9.96	0.60 to 0.89	0.79 to 1.07		1.12 to 1.69
Estonia											
RR	0.17*		0.93		1.12*	0.16*	5.14*	0.31*	uc†		1.35
95% CI	0.10 to 0.27		0.49 to 1.78		1.05 to 1.20	0.06 to 0.46	2.48 to 10.74	0.18 to 0.44			0.78 to 2.35
Finland											
RR	0.34*		4.91*		1.61*	0.38*	11.68*	0.72*	0.79		1.53*
95% CI	0.32 to 0.37		3.15 to 7.67		1.45 to 1.79	0.31 to 0.47	8.37 to 16.33	0.58 to 0.90	0.63 to 1.00		1.16 to 2.03
Germany											
RR	0.35*		1.32*		1.45*	0.29*	8.02*	0.55*	1.06		0.85*
95% CI	0.34 to 0.37		1.20 to 1.45		1.42 to 1.48	0.27 to 0.32	7.03 to 9.15	0.52 to 0.58	0.99 to 1.14		0.80 to 0.90
Hungary											
RR	0.26*		0.77*		1.54*	0.31*	7.39*	0.52*	1.08		1.11
95% CI	0.24 to 0.28		0.66 to 0.89		1.48 to 1.60	0.24 to 0.39	4.70 to 11.64	0.45 to 0.59	0.85 to 1.38		0.95 to 1.31
Iceland											
RR	0.50*		0.60		2.20*	0.38*	uc†	1.97	uc†		1.15
95% CI	0.32 to 0.81		0.25 to 1.51		1.14 to 4.56	0.15 to 0.95		0.33 to 12.35			0.29 to 4.77
Ireland											
RR	0.28*		2.31*		1.80*	0.63*	4.00*	0.51*	0.41*		0.98
95% CI	0.23 to 0.35		1.03 to 5.21		1.58 to 2.06	0.53 to 0.76	2.09 to 7.68	0.29 to 0.89	0.25 to 0.67		0.42 to 2.31
Italy											
RR	0.36		1.25		1.92*	0.41*	4.46	0.42*	0.30		1.63
95% CI	0.12 to 1.08		0.52 to 3.13		1.33 to 2.92	0.18 to 0.96	0.79 to 26.31	0.26 to 0.68	0.08 to 1.07		0.42 to 6.51
Luxembourg											
RR	0.34*		0.90		2.56*	1.19	13.97*	0.52*	0.76		0.91
95% CI	0.21 to 0.54		0.45 to 1.86		1.60 to 4.20	0.37 to 3.93	2.52 to 80.14	0.36 to 0.76	0.22 to 2.66		0.47 to 1.80
Netherlands											
RR	0.44*		1.50*		1.43*	0.62*	8.67*	0.76*	0.97		1.14
95% CI	0.40 to 0.49		1.16 to 1.94		1.35 to 1.52	0.54 to 0.72	5.11 to 14.74	0.67 to 0.87	0.86 to 1.10		0.96 to 1.36
Portugal											
RR	0.23*		0.61*		1.66*	0.33*	4.63*	0.49*	0.83		0.98
95% CI	0.17 to 0.31		0.53 to 0.70		1.52 to 1.82	0.27 to 0.41	3.24 to 6.66	0.39 to 0.60	0.47 to 1.48		0.79 to 1.22
Scotland											
RR	0.38*		6.15*		1.69*	0.60*	19.47*	0.89	1.55		1.08
95% CI	0.34 to 0.42		3.31 to 11.47		1.53 to 1.86	0.44 to 0.82	3.41 to 111.63	0.66 to 1.19	0.90 to 2.68		0.78 to 1.48
Slovenia											
RR	0.26*		1.43		1.19*	0.19*	11.62*	0.39*	0.42*		0.91
95% CI	0.18 to 0.38		0.99 to 2.05		1.10 to 1.28	0.14 to 0.27	5.63 to 24.13	0.29 to 0.54	0.25 to 0.70		0.62 to 1.34
Spain											
RR	0.41*		0.68*		1.79*	0.57*	7.19*	0.49*	1.25*		0.86*
95% CI	0.36 to 0.47		0.60 to 0.78		1.70 to 1.87	0.50 to 0.65	5.38 to 9.60	0.46 to 0.51	1.04 to 1.49		0.77 to 0.96
Switzerland											
RR	0.35*		1.46*		1.42*	0.30*	9.37*	0.62*	0.70*		1.07
95% CI	0.32 to 0.38		1.07 to 2.00		1.29 to 1.57	0.24 to 0.36	7.48 to 11.74	0.54 to 0.71	0.56 to 0.84		0.84 to 1.36
Total											
RR	0.35*		1.26*		1.53*	0.39*	7.22*	0.54*	0.93		0.94
95% CI	0.33 to 0.37		1.12 to 1.43		1.48 to 1.59	0.35 to 0.44	5.91 to 8.82	0.50 to 0.58	0.83 to 1.04		0.85 to 1.04

*p<0.05; †uncalculable.

Females more often used poisoning by drugs than males in all countries. It was only in Italy that the higher risk of female poisoning by drugs was not statistically significant. Similarly, drowning was more common in females than in males in all countries except in Luxembourg. Females also had a higher risk of jumping than males, except in Iceland. Other suicide methods varied by country (table 3).

Grouping with hierarchical clustering using the Ward method showed that countries might be divided into five main groups by suicide methods among males. The first group (Estonia, Slovenia, Hungary) had a very high proportion of hanging and low proportions of drowning and using a moving object as a mean. The second group (Finland and Switzerland) had the highest proportion of using firearms and the lowest of hanging in comparison with other countries; also poisoning by drugs was above average. The third group (Germany, Belgium and The Netherlands) had average proportions of hanging and poisoning by drugs. In countries in the fourth group (England, Scotland and Iceland), the proportion of poisoning by drugs was higher than in other countries and poisoning with other means was above average. The fifth group (Italy, Spain, Luxembourg and Portugal) had a high proportion of jumping along with a low proportion of using a moving object. Italy, Portugal and Spain also had low percentages of poisoning by drugs. Ireland could not be grouped with any other countries owing to its high proportion of drowning.

For females, grouping with hierarchical clustering divided countries into three triplets and three pairs. The first cluster consisted of England, Scotland and Hungary which all had high proportions of poisoning by drugs, low percentages of jumping and below average proportions of drowning. The second cluster, Germany, Slovenia and The Netherlands, was similar to the overall average. Estonia had a very high proportion of hanging and very low proportions of drowning and use of a moving object, consequently Estonia could not be grouped with any other countries. Finland and Switzerland each had high percentages of poisoning by drugs and low proportions of hanging. Belgium and Ireland had high proportions of drowning and low proportions of jumping. Italy, Luxembourg and Spain had very high proportions of jumping and low proportions of hanging. The last pair, Iceland and Portugal, ranked above average in drowning and low in the use of a moving object. Hanging was ranked below average.

## DISCUSSION

### Methodological considerations

The present study is, to our knowledge, the first multisite epidemiological examination of X-coded deaths in Europe describing, comparing and analysing the pattern of the seven most frequent suicide methods by sex in 16 European countries representing 308 319 445 inhabitants, or 52% of the European population (Commonwealth of Independent States not included; http://data.euro.who.int/hfamdb/). The data reported is based on the average of five to six recent years. This reporting scheme eliminates temporary fluctuations essential to small numbers characteristic of suicide mortality, particularly when divided by gender and suicide methods.

One of the limitations of the study was that two different classifications of causes of death were covered: in England, Ireland and Portugal the 9th revision of the ICD was used, which was replaced by X-coded death using ICD-10 in the other countries.

To verify that the EAAD sample could represent the whole of Europe in terms of suicide mortality, the average annual age-adjusted suicide rates between the years 2000–5 were compared. For EAAD countries combined suicide rates 16.3 for 100 000 males and 5.0 for 100 000 females was lower against suicide mortality of the whole of Europe (Commonwealth of Independent States included)—28.9 for males and 6.1 for females. The respective age-adjusted rates for 15 European Union member states (until 1 May 2004)—16.0 for males and 5.0 for females (average for 2000–4)—were similar to that of the average of the EAAD countries combined.

With respect to individual countries, there were several countries that were limited by non-representative samples: Belgium was represented only by the Flemish region and Italy was represented only by South Tyrol County. Also, two regions were reported separately in the United Kingdom. As England and Scotland are two separate sites in the EAAD study, they are reported here with caution as two separate countries.

To test the reliability of data obtained by EAAD partner countries, male and female suicide numbers were compared to WHO data in the European Mortality Database. There was agreement between the WHO data and the EAAD compiled data in Estonia, Finland, Germany, Hungary, The Netherlands, Portugal, Slovenia and Spain. For cites where a region, rather than the whole country was studied (Belgium, Italy, England and Scotland), data were not comparable with WHO data. There were some notable differences between EAAD and WHO data. The EAAD data for Ireland was 8.5% and for Luxembourg 1.8% higher than the WHO data. In contrast, the EAAD data from Iceland and Switzerland were lower—2.9% and 0.5% respectively.

### Male and female suicide methods

Although completed suicide rates are generally much higher in men than in women with the exception of some Asian countries,^15^^–^17 there are more suicide attempts registered for females by the WHO/EURO Multicentre Study in most of the participating countries,^16^ and much more diagnosed depression in women relative to men.^10^ ^12^ ^18^ This gender paradox calls into question the possible reasons for different prevalence rates in male-female completed suicides.^9^ ^19^

Several studies have addressed the issue of different patterns of methods used in male and female suicide populations as the primary reason for gender differences in suicide mortality. Traditionally, women have selected suicide methods that are less lethal and men have chosen techniques that are more violent and whose consequences are irreversible.^3^–^5 20^

Spicer *et al*^7^ revealed that firearms, drowning and suffocation/hanging were the most lethal methods and drug overdose/poison ingestion and cutting/piercing were the least lethal methods. In Card’s classification,^21^ in addition to the above mentioned methods jumping from a high place and jumping in front of a moving object were added to the highly lethal methods. Card qualified the use of fire as a less lethal suicide method.

The present study found hanging to be the most predominant method of suicide in all EAAD countries combined. In fact, 54.3% of males and 35.6% of females in our study died from hanging. As hanging is universally available, it makes sense that it is the most common suicide method in many countries worldwide^22^; however, there is considerable variability internationally. A study of suicide methods in a large number of cases in Japan and the United States revealed that Japan had a very high proportion of hanging (70.4% for males and 60% for females); this proportion was much lower (18.2% for males and 16.2% for females) in the United States.^23^ Similarly, an Australian study reported hanging in 32% of its cases.^24^ Hanging and self-poisoning with pesticides were the preferred means of suicide in south India.^25^

Self-poisoning (X60–X69) ranked as the second highest suicide method for both males (14.0%) and females (29.0%) in EAAD countries combined. In the EAAD this usually meant poisoning by drugs—that is, medication (X60–X64), in contrast to worldwide trends, which show that self-poisoning by pesticides is the frequently used method among other poisonings (X65–X69) in many Asian, African and Latin American countries, accounting in rural areas for about a third of all suicides worldwide.^1^ In fact, in rural China pesticide ingestion accounts for 58% of all suicides and accounts for 79% of all suicides among young females in rural areas.^26^

Firearms, a highly lethal method, ranked the third among males (9.7%) and were rarely used among females (1.3%) in EAAD countries combined. In the United States, respective proportions were much higher—63.1% and 37.2%.^23^ An Australian study^24^ reported the use of firearms in 22% of total suicides. In Japan^23^ and China^27^ the use of firearms as a means of suicide was rare. The literature on this topic shows that limiting access to firearms has been found to be an effective mean of reducing suicide mortality.^28^ ^29^

Notwithstanding the similar rank order of suicide methods among males and females combined, males had a 7.2 times higher risk of using firearms and a 1.5 times higher risk of hanging than females, while poisoning by drugs and drowning were methods predominated by females. Remaining methods had different male-female dominance in different countries. Considering the classification systems of Spicer *et al*^7^ and Card,^21^ in European countries males had a higher risk of using more lethal methods like hanging and firearms than females.

### Grouping countries

There was a strong similarity between Hungary, Slovenia and Estonia, based on very high shares of hanging both for males and females. In an effort to find an explanation, we note the following common features: recent socialist backgrounds, rapid socio-political and economic changes in integrating into the European Union, very high alcohol consumption, high rates of alcohol dependence and abuse among suicidents^17^ ^30^^–^33 and high overall suicide rates.

Referring to the cluster analysis of male suicide methods, excluding the former Eastern block countries (Estonia, Hungary and Slovenia) and Finland and Switzerland, the remaining groups of countries appear, at the first glance, to be similar in suicide methods by geographic proximity. In northwest Europe (England, Scotland and Iceland), high proportions of poisoning by drugs were found. Central Europe (Belgium, Germany and The Netherlands) was close to the EAAD average concerning most methods and southern Europe (Italy, Portugal, Spain, including also Luxembourg) was characterised by a very high proportion of jumping. Ireland could not be grouped because of the extremely high level of drowning. It was surprising to find an almost congruent pattern of suicide methods for both genders in Switzerland and Finland characterised by high rates of poisoning by drugs and firearms and also low proportions of hanging. Such commonalities were not present in females and female clusters were not identifiable.

### Prevention possibilities

The high rate of hanging places considerable limitations on the reduction of suicides through method restriction except in institutional settings.^34^ ^35^ De Leo *et al*^24^ associated hanging with having low impulse control, being in short-term, suicidal crisis and receiving past or current psychiatric treatment. They called for further investigations to develop a deeper understanding of hanging and to develop appropriate prevention methodologies. Cantor and Baume^34^ also pointed out that little research has examined hanging and suggested hanging might be influenced by changing public perceptions of the social acceptability of suicide by hanging.

Self-poisoning, as a potentially preventable method,^28^ ^36^ should be the topic of further investigations regarding the promotion of suicide prevention policy. X-classification offers the opportunity to assess the use of self-poisoning as a suicide method. A cross-sectional global public health initiative with the overall goal of reducing morbidity and mortality related to pesticide poisoning has been announced by the WHO.^1^ Self-poisoning by drugs accounts for one fourth of female deaths in EAAD countries and in some of the countries almost a half of female suicides. As such, self-poisoning deserves attention and the application of best practices in the field.

There are remarkably few papers on suicide prevention addressing gender issues on method choice.^37^ Identifying and comparing patterns of methods of suicides used in different countries by gender could lead to the development of intervention strategies. The present study identifies groups of countries with similar patterns of suicide methods by gender and encourages the research and development of collaborative suicide prevention strategies. Nevertheless, control of access to methods is but one of the strategies for suicide prevention. Other approaches, such as better identification and treatment of mental disorders and the improvement of toxicological aid, should be put in place.

Further analysis of other sociodemographic correlates is warranted, particularly the age distribution of the utilisation of suicide methods and a detailed analysis of poisoning by drugs as a preventable method to complete suicide.

**What is already known on this subject**

- Two types of interventions by comprehensive overview of Mann *et al*^2^ were proved to be evidence based in suicide-preventive interventions: educating physicians to recognise and treat depression and restricting access to lethal means.
- From studies of the previous literature choice of method differs by gender and is influenced by availability, lethality and acceptability.
- To our knowledge there are no recent data published for Europe on suicide methods.
- Analysis of methods is needed to elaborate effective collaborative suicide prevention strategies.

**What this study adds**

- The database of the present study contains unique information about suicide methods employed during recent years in 15 countries covering 52% of the European population.
- This paper, the first in a series of articles in preparation, gives a gender-specific overview and compares suicide methods used in total and cross-nationally.
- In the European countries studied combined hanging and firearms were most prevalent for male suicides, while hanging and poisoning by drugs was common for females.
- However, there were differences in the distribution of suicide methods between the countries and groups of countries, which enhances our understanding of suicide and provides the opportunity to develop prevention strategies.

**Policy implications**

Research on suicide methods, taking into account best practices in restriction of means, could lead to the development of effective intervention strategies.

## References

[1] Bertolote JM Fleischmann A Eddleston M Deaths from pesticide poisoning: a global response. Br J Psychiatry 2006;189:201–3 16946353 10.1192/bjp.bp.105.020834 PMC2493385

[2] Mann JJ Apter A Bertolote J Suicide prevention strategies—a systematic review. JAMA 2005;294:2064–74 16249421 10.1001/jama.294.16.2064

[3] McIntosh J Methods of suicide. Maris R, Berman A, Maltsberger J, *et al.*, Assessment and prediction of suicide. New York, London: The Guilford Press, 1992:382–97

[4] Denning DG Conwell Y King D Method choice, intent, and gender in completed suicide. Suicide Life Threat Behav 2000;30:282–8 11079640

[5] Hawton K Sex and suicide. Gender differences in suicidal behaviour. Br J Psychiatry 2000;177:484–5 11102320 10.1192/bjp.177.6.484

[6] Rich CL Ricketts JE Fowler RC Some differences between men and women who commit suicide. Am J Psychiatry 1988;145:718–22 3369559 10.1176/ajp.145.6.718

[7] Spicer RS Miller TL Suicide acts in 8 states: incidence and case fatality rates by demographics and method. Am J Public Health 2000;90:1885–91 11111261 10.2105/ajph.90.12.1885 PMC1446422

[8] Hendin H Suicide in America. New York: Norton, 1982

[9] Canetto S Sakinofsky I The gender paradox in suicide. Suicide Life Threat Behav 1998;28:1–23 9560163

[10] Moscicki E Gender differences in completed and attempted suicides. Ann Epidemiol 1994;4:152–1 8205283 10.1016/1047-2797(94)90062-0

[11] Lester D Why do people choose particular methods for suicide. Act Nerv Super 1988;30:312–4 3227770

[12] Murphy GE Why women are less likely than men to commit suicide. Compr Psychiatry 1998;39:165–75 9675500 10.1016/s0010-440x(98)90057-8

[13] Hegerl U Wittman M Arensman E The European Alliance Against Depression (EAAD): a multifaceted, community based action programme against depression and suicidality. World J Biol Psychiatry 2008;9:51–81 17853299 10.1080/15622970701216681

[14] Hegerl U Althaus D Schmidtke A The alliance against depression: 2-year evaluation of a community-based intervention to reduce suicidality. Psychol Med 2006;36:1225–33 16707028 10.1017/S003329170600780X

[15] Schmidtke A Weinacker B Apter A Suicide rates in the world: update. Arch Suicide Res 1999;5:81–9

[16] Schmidtke A Weinacker B Löhr C Suicide and suicide attempts in Europe—an overview. In: Schmidtke A Bille-Brahe U De Leo D, eds. *Suicidal behaviour in* Europe. Results from the WHO/EURO Multicentre Study on Suicidal Behaviour. Seattle, Toronto, Bern, Gottingen: Hogrete & Huber, 2004:15–28

[17] Bertolote JM Suicide in the world: an epidemiological overview, 1959–2000. Wasserman D, ed, ed. Suicide—an unnecessary death. London: Martin Dunitz, 2001:3–10

[18] Wålinder J Rutz W Male depression and suicide. Int Clin Psychopharmacol 2000;16:21–4 10.1097/00004850-200103002-00004 11349757

[19] Moller-Leimkuhler AM The gender gap in suicide and premature death or: why are men so vulnerable? Eur Arch Psychiatry Clin Neurosci 2003;253:1–8 12664306 10.1007/s00406-003-0397-6

[20] Marušic A Landau S Tomori M Long-term trends, seasonality, weekly distribution, and methods of suicide in Slovenia: a comparison between the younger and older population. Arch Suicide Res 2003;7:135–43

[21] Card JJ Lethality of suicidal methods and suicide risk: two distinct concepts. Omega 1974;5:37–45

[22] Lester D Changes in the methods used for suicide in 16 countries from 1960–1980. Acta Psychiatr Scand 1990;81:260–1 2343750 10.1111/j.1600-0447.1990.tb06492.x

[23] Ojima T Nakamura Y Detels R Comparative study about methods of suicide between Japan and the United States. J Epidemiol 2004;14:187–92 15617392 10.2188/jea.14.187 PMC8784240

[24] De Leo D Evans R Neulinger K Hanging, firearm, and non-domestic gas suicides among males: a comparative study. Aust N Z J Psychiatry 2002;36:183–9 11982538 10.1046/j.1440-1614.2001.01013.x

[25] Bose A Konradsen F John J Mortality rate and years of life lost from unintentional injury and suicide in south India. Trop Med Int Health 2006;11:1553–6 17002729 10.1111/j.1365-3156.2006.01707.x

[26] Yang GH Phillips MR Zhou MG Understanding the unique characteristics of suicide in China: national psychological autopsy study. Biomed Environ Sci 2005;18:379–89 16544520

[27] He ZX Lester D Methods for suicide in mainland China. Death Studies 1998;22:571–9 10342941 10.1080/074811898201407

[28] Leenaars A Cantor C Connolly J Controlling the environment to prevent suicide: international perspectives. Can J Psychiatry 2000;45:639–44 11056826 10.1177/070674370004500706

[29] Shenassa ED Catlin SN Buka SL Lethality of firearms relative to other suicide methods: a population based study. J Epidemiol Community Health 2003;57:120–4 12540687 10.1136/jech.57.2.120 PMC1732374

[30] Kolves K Varnik A Tooding LM The role of alcohol in suicide: a case-control psychological autopsy study. Psychol Med 2006;36:923–30 16650347 10.1017/S0033291706007707

[31] Varnik A Kolves K Vali M Do alcohol restrictions reduce suicide mortality? Addiction 2007;102:251–6 17222279 10.1111/j.1360-0443.2006.01687.x

[32] Zonda T One-hundred cases of suicide in Budapest: a case-controlled psychological autopsy study. Crisis 2006;27:125–9 17091822 10.1027/0227-5910.27.3.125

[33] Bilban M Skibin L Presence of alcohol in suicide victims. Forensic Sci Int 2005;147:9–12 15694738 10.1016/j.forsciint.2004.09.085

[34] Cantor CH Baume PJM Access to methods of suicide: what impact? Aust N Z J Psychiatry 1998;32:8–14 9565178 10.3109/00048679809062700

[35] Ohberg A Trends and availability of suicide methods in Finland. Proposals for restrictive measures. Br J Psychiatry 1995;166:35–43 7894873 10.1192/bjp.166.1.35

[36] Pirkola S Isometsa E Lonnqvist J Do means matter? Differences in characteristics of Finnish suicide completers using different methods. J Nerv Ment Dis 2003;191:745–50 14614342 10.1097/01.nmd.0000095127.16296.c1

[37] Beautrais A Gender issues in youth suicidal behaviour. Emerg Med 2002;14:35–42 10.1046/j.1442-2026.2002.00283.x 11993833

